# Using an Improved Phagocytosis Assay to Evaluate the Effect of HIV on Specific Antibodies to Pregnancy-Associated Malaria

**DOI:** 10.1371/journal.pone.0010807

**Published:** 2010-05-25

**Authors:** Ricardo Ataíde, Wina Hasang, Danny W. Wilson, James G. Beeson, Victor Mwapasa, Malcolm E. Molyneux, Steven R. Meshnick, Stephen J. Rogerson

**Affiliations:** 1 Department of Medicine, University of Melbourne, Post Office Royal Melbourne Hospital, Melbourne, Victoria, Australia; 2 GABBA, Graduate Program in Areas of Basic and Applied Biology, Universidade do Porto, Porto, Portugal; 3 Infection and Immunity Division, the Walter and Eliza Hall Institute of Medical Research, Melbourne, Victoria, Australia; 4 Malawi-Liverpool-Wellcome Trust Clinical Research Programme, College of Medicine, University of Malawi, Blantyre, Malawi; 5 Department of Community Health, University of Medicine, University of Malawi, Blantyre, Malawi; 6 School of Tropical Medicine, University of Liverpool, Liverpool, United Kingdom; 7 Department of Microbiology and Immunology, University of North Carolina, Chapel Hill, North Carolina, United States of America; Université Pierre et Marie Curie, France

## Abstract

**Background:**

Pregnant women residing in malaria endemic areas are highly susceptible to *Plasmodium falciparum* malaria, particularly during their first pregnancy, resulting in low birth weight babies and maternal anaemia. This susceptibility is associated with placental sequestration of parasitised red blood cells expressing pregnancy-specific variant surface antigens. Acquisition of antibodies against these variant surface antigens may protect women and their offspring. Functions of such antibodies may include prevention of placental sequestration or opsonisation of parasitised cells for phagocytic clearance.

**Methodology/Findings:**

Here we report the development and optimisation of a new high-throughput flow cytometry-based phagocytosis assay using undifferentiated Thp-1 cells to quantitate the amount of opsonizing antibody in patient sera, and apply this assay to measure the impact of HIV on the levels of antibodies to a pregnancy malaria-associated parasite line in a cohort of Malawian primigravid women. The assay showed high reproducibility, with inter-experimental correlation of r^2^ = 0.99. In primigravid women, concurrent malaria infection was associated with significantly increased antibodies, whereas HIV decreased the ability to acquire opsonising antibodies (Mann-Whitney ranksum: *p = *0.013). This decrease was correlated with HIV-induced immunosuppression, with women with less than 350×10^6^ CD4+ T- cells/L having less opsonising antibodies (coef: −11.95,P = 0.002). Levels of antibodies were not associated with protection from low birth weight or anaemia.

**Conclusions/Significance:**

This flow cytometry-based phagocytosis assay proved to be efficient and accurate for the measurement of Fc-receptor mediated phagocytosis-inducing antibodies in large cohorts. HIV was found to affect mainly the acquisition of antibodies to pregnancy-specific malaria in primigravidae. Further studies of the relationship between opsonising antibodies to malaria in pregnancy and HIV are indicated.

## Introduction

In malaria-endemic areas, pregnancy increases susceptibility to infections by the malaria parasite *Plasmodium falciparum*. Pregnant women with *P. falciparum* malaria are more likely to have low birth weight (LBW) babies and to suffer from anaemia, especially during their first pregnancy (reviewed in [1], [2], [3]). During pregnancy the placenta expresses and exposes to the maternal blood circulation chondroitin sulphate A (CSA) and this is one of the favoured receptors for the binding of red cells infected with parasites expressing pregnancy-associated variant surface antigens (VSA) [4]. These VSA are parasite derived proteins expressed on the surface of the parasitized red blood cells (pRBC). *P. falciparum* erythrocyte membrane protein 1 (PfEMP1) is the most extensively studied of the VSA, and acts as a major mediator of the parasite sequestration and immune evasion that characterise *P. falciparum* infections (reviewed in [5]). Identifying the determinants of immunity to malaria in pregnancy is critical to understanding the pathogenesis of the disease, and having a reliable and convenient measure of protection from infection in pregnant women who live in endemic regions is an important goal for local and international public health authorities. The currently most favoured measure of protection is antibodies that are directed against pregnancy specific parasites. It is thought that the acquisition of antibodies against VSA expressed on the surface of pRBC, over successive pregnancies, may protect the women and their offspring [6], [7]. In areas where HIV and malaria co-exist, higher rates of malaria infection, higher densities and prevalence of parasitaemia and lower levels of antibodies to pregnancy-specific VSA are associated with HIV infection making the combined presence of both HIV and malaria particularly deleterious for the health of both mothers and newborns [8], [9], [10], [11], [12].

Antibodies to pregnancy-associated VSA have previously been measured by agglutination assays, anti-adhesion assays or assays measuring IgG antibodies to pregnancy-associated VSA; more recently the ability of these antibodies to induce phagocytic clearance of pRBC (phagocytic antibodies) has been measured (reviewed in [13], [11], [14]). Antibodies that specifically contribute to the phagocytosis of opsonised pRBC were shown to be decreased in the serum of HIV-positive women [15].

Undifferentiated Thp-1 cells (uThp-1) are pro-monocytic cells [16], shown to phagocytose IgG covered particles through Fcγ receptors [17]. The phagocytic response by Thp-1 cells correlates with serum titres of IgG against VSA [14], [18] and models using adherent, chemically-differentiated Thp-1 cells (dThp-1) are useful in evaluating antibodies as measures of protection in pregnant women [11], [14] but these assays are time-consuming and destroy the effector cells. Also, in contrast to uThp-1, dThp-1 express receptors such as CD36 that are able to promote non-Fc-receptor mediated phagocytosis [19], [20].

Here we present a new, easier and more high-throughput Thp-1 assay, using uThp-1 cells and flow cytometry. By performing this new assay in parallel with an assay measuring the total levels of VSA-specific IgG present in the serum, we determined the impact of HIV on levels and function of antibodies towards the pregnancy specific CSA-binding parasite-line CS2 in a cohort of primigravid women.

## Methods

### Ethics statement

Ethical clearance for the study was provided by the College of Medicine Research Ethics Committee, University of Malawi, and the Melbourne Health Human Research Ethics Committee.

### Study samples

The serum samples used in this assay came from a cohort which has previously been described [11], [21]. In brief, women in late third trimester of pregnancy consented to studies including HIV testing, and samples of peripheral blood were collected. Only primigravid women were included in the present study, resulting in a total of 263 samples analysed.

Placental tissue, collected at delivery, was fixed in formalin and Giemsa stained sections were examined histologically as previously described [22]. Placental malaria was considered to be present if there was evidence of parasites or pigmented monocytes or pigment in fibrin deposits in the placenta, irrespective of the presence of parasites in the peripheral blood (unless stated otherwise).

We combined histology findings with results of microscopy of Giemsa-stained placental and peripheral blood thick films to allocate women to one of four groups: *No Malaria* (no evidence of past or current malaria, either in blood films or placental histology), *Acute Infection* (pRBC on placental histology, without malaria pigment deposition), *Chronic Infection* (both pRBC and malaria pigment deposits on histology) and *Past Infection* (placental malaria pigment deposits, without pRBC on histology or on blood films). We excluded from the grouping analysis women who presented only with peripheral parasitaemia. All infections were with *P. falciparum*.

Maternal anaemia (anaemia) was defined as a haemoglobin measurement of less than 11.0 g/dL. Infant low birth weight (LBW) was considered to be a baby weight of less than 2,500 g.

### Cell culture and Parasites

Thp-1 cells were maintained in RPMI 1640 (GIBCO) supplemented with 10% heat-inactivated Foetal Bovine Serum (FBS, GIBCO), 1% penicillin-streptomycin (GIBCO), 2 mM Glutamine (GIBCO), 25mM HEPES (GIBCO) and 55×10^−3^ mM 2-Mercaptho-Ethanol (SIGMA) at a density below 5×10^5^ cells/mL. THP-1 cells were obtained from the ATCC (catalog number: TIB-202™). THP-1 pro-monocytic cells were originally derived from a patient with monocytic leukemia [16]. Undifferentiated THP-1 cells used in our assays had not been exposed to agents such as PMA (phorbol-12-myristate-13-acetate) or TPA (12-O-tetradecanoylphorbol-13-acetate) [23], [24].

The CS2 parasite line is recognised as being similar to placental-type isolates and known to bind to CSA and to be recognised by serum in a pregnancy-specific and gravidity-specific manner [25], [26]. The parasite lines CS2 and CS2 transfected with a plasmid containing a green fluorescent protein (GFP) expression cassette (Wilson et al. in preparation) were maintained in RPMI-HEPES supplemented with 0.5% AlbumaxII (GIBCO) and 25mM of NaHCO_3_ (parasite medium) and gelatin selected (by flotation) every 2 weeks. The GFP expression plasmid was maintained episomally by addition of 4ug/ml of Blasticidin-S-HCL (Invitrogen) to CS2-GFP in continuous culture. GFP expression was confirmed by staining pRBC cultures with 10 µg/mL of Ethidium Bromide (EtBr, Bio-Rad Laboratories) for 15 min at room temperature and comparing EtBr fluorescence in the FL-2 channel with that of GFP in the FL-1 channel using a FACSCalibur flow cytometer (BD Biosciences).

### Assays of IgG to CS2 VSA

IgG to CS2 VSA was measured as described by Mount and colleagues [21] with minor modifications. In summary mid to late trophozoite-stage CS2 pRBC at 3–10% parasitaemia were washed thrice in PBS/1% newborn calf serum (PBS/NCS) by centrifuging at 400 g for 5 minutes, resuspended at 0.1% haematocrit in PBS/NCS, and incubated with test serum at 1/20 dilution for 30 minutes in a 96-well plate at RT. Cells were washed thrice. Rabbit anti-human IgG (Dako) 1/100 in PBS/NCS was added and incubated for 30 minutes at RT. After washing as before, Alexafluor 488-conjugated donkey anti-rabbit IgG (Invitrogen) was added at 1/500 dilution in PBS/NCS containing 10 µg/ml EtBr and samples were incubated in the dark for 30 minutes at RT. Cells were washed once again and resuspended in 150 µl PBS before being analysed on a FACSCalibur flow cytometer. BD CellQuest™ software version 5.2.1 (BD Biosciences) was used to acquire and analyse the data.

The RBC population was gated based on FSC and SSC. CS2 pRBC were recognized by EtBr fluorescence and 1500 EtBr positive cells were acquired. The FL-1 Mean Fluorescence Intensity (MFI) above negative controls was used as a relative measure of human IgG binding to pRBCs for each serum sample. Samples were run in duplicate. Positive control was a pool of serum with known high antibody recognition to CS2. Negative controls were from six unexposed Australian donors.

### The phagocytosis Assay

#### a) The parasites

Mid- to late-trophozoite stage CS2-GFP pRBC were purified by density gradient centrifugation. pRBC were overlaid on a gradient of 80%, 60% and 40% Percoll (Amersham) in parasite medium. After centrifugation at 2,000 g×15 min, cells resting on the 60% layer were collected and washed thrice at RT with RPMI-HEPES (400 g for 3 min). Cells were resuspended at 5×10^7^/mL. In a 96-well u-bottom plate previously coated with FBS, 3.3 µL of serum samples and dilutions of a standard patient pooled serum (pps) were incubated with 30 µL of pRBC suspension and mixed. This included one well with no serum sample (no-serum control) and one well with no parasite (Thp-1 alone control). pRBC were opsonised by the serum for 1 h at RT, in the dark and resuspended once, within that hour. The cells were then washed thrice as before and resuspended in 150 µL of Thp-1 medium. Following this 50 µL were aliquoted into three wells of a final 96-well u-bottom plate.

#### b) Thp-1 cells

While the pRBC were being opsonised, Thp-1 cells were collected, centrifuged at 300 g for 5 min at RT and resuspended at 5×10^5^/mL in Thp-1 medium.

#### c) Phagocytosis

One hundred microliters of the Thp-1 suspension were dispensed into each well of the final plates which contained 50 µL pRBC suspension. The cells were mixed and left to phagocytose for 40 min at 37°C in a 5% CO_2_ humidified incubator. Phagocytosis was stopped by centrifugation at 4°C, 350 g for 3 min. The supernatant was discarded and the cells were resuspended at 37°C in 150 µL of FACS Lysing solution (BD Biosciences) at a 1/10 dilution in distilled water, and left at RT for 10 min in order to eliminate unphagocytosed pRBC. Lysis was stopped by the addition of 50 µL of cold PBS (−Ca^2+^, −Mg^2+^) 2% FBS and 0.02% NaNO_3_ (FACS Buffer). The plates were centrifuged at 4°C, 350 g×3 min. After 3 washes with 150 µL of cold FACS Buffer at 4°C, 350 g×3 min, the cells were fixed in cold 2% Paraformaldehyde in PBS and left on ice before acquiring.

#### d) Flow Cytometry

Cells were acquired in a FACSCalibur flow cytometer. Thp-1 cells alone were first gated on the basis of their forward- and side-scatter properties. Their level of auto-fluorescence in the FL-1 channel was set to always be less than 5%. Sample triplicates were then acquired, usually at a rate of 250–350 cells/s until 10,000 Thp-1 cells were acquired.

#### e) The analysis

All results were analysed using BD CellQuest™ software version 5.2.1. Triplicate results for samples and standard curves were obtained after subtraction of the “Thp-1 alone” results. Using GraphPad Prism version 4.2 for Windows (GraphPad Software, San Diego California USA), the means of the triplicate standard curve phagocytosis results were plotted against their dilution. The result was a saturation curve. After natural log transformation of the x-axis, a standard curve was obtained. Samples were then transformed in the same way, plotted on the standard curve and transformed back in order to obtain their relative titre.

**Figure 1:**
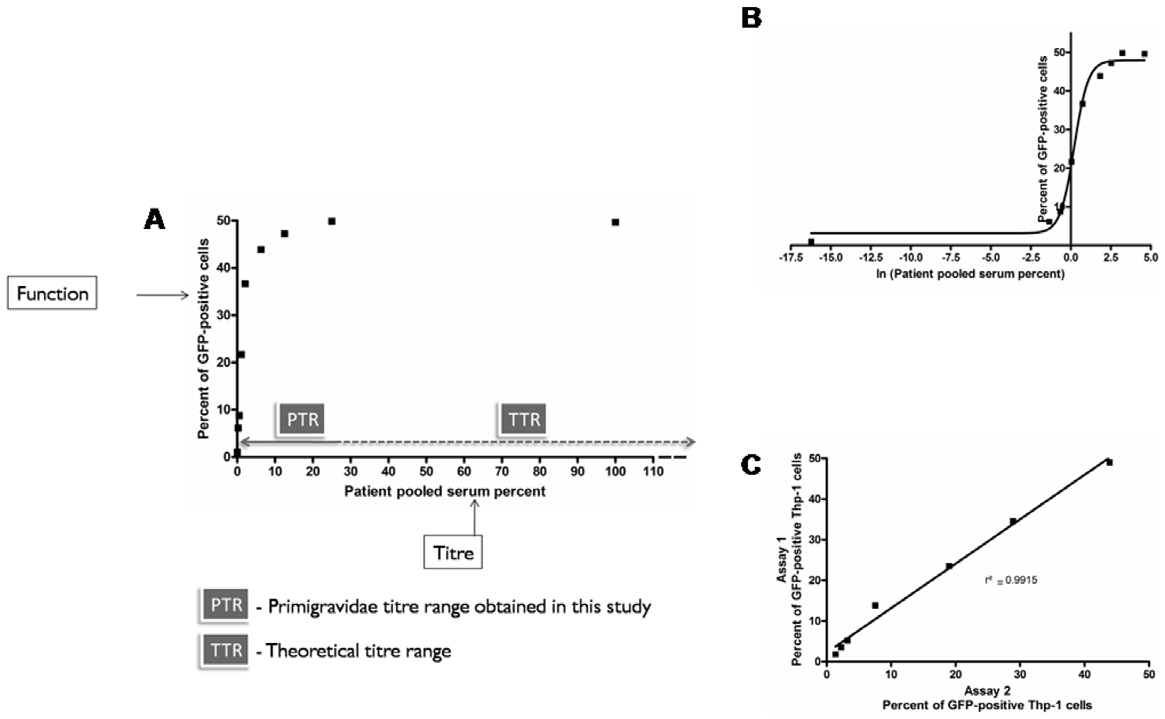
Phagocytosis profile of a typical uThp-1 phagocytosis assay standard curve and inter-assay reproducibility. A) Typical standard curve plot of percentage of patient pooled serum against percent of GFP-positive uThp-1 cells with indications of absolute phagocytosis values used for phagocytic antibodies' function, titre and both the titre range obtained in this study (PTR) and a theoretical titre range (TTR) (extrapolated from the patient pooled serum titre). B) Typical transformation of standard curve results. X-axis is the natural logarithm (ln) of the patient pooled serum. Goodness of fit: r^2^ = 0.99. For all standard curves r^2^ was always higher than 0.97. C) Correlation of results obtained on different experimental days. CS2 –GFP RBC opsonised with increasing dilutions of pooled patient serum were incubated with uThp-1 cells for 40 min and phagocytosis was measured by flow cytometry. The absolute phagocytosis values obtained on both experimental days are plotted. A dilution of 1/10 of original serum was always considered as the 100%. No-serum control would be assigned an arbitrary value of 9×10^−8^%. uThp-1 cells alone were considered absolute zero. Analysis performed on GraphPad Prism v4.0

Two parameters were obtained when using this assay:

1. The phagocytic antibodies' function, measuring the percentage of Thp-1 cells that phagocytosed pRBC compared to the maximum percentage obtained in the standard curve (e.g. a result of 30% Thp-1 cells positive on a sample compared to 60% Thp-1 cells positive using positive control serum would give a 50% phagocytic activity);
2. The phagocytic antibodies' titre, which like an ELISA, compares the test sample to the standard curve of the positive control to derive a relative titre of antibody (figure 1A and 1B).

Phagocytosis results higher than the standard curve, by the nature of the assay, were discarded from the analysis.

### Database analysis

Results for all the assays performed were combined in Stata v9.2 (StataCorp. 2005. *Stata Statistical Software: Release 9*. College Station, TX: StataCorp LP), with a database containing the clinical information on the study participants, and were statistically analysed for the relevant study parameters. In some instances, stated in the text, GraphPad Prism v 4.2 was used for statistical analyses.

Age, birth weight and maternal haemoglobin levels were all normally distributed and Student's t-tests were applied. Total IgG to CS2 VSA, and phagocytic antibodies were not normally distributed and were analysed using Mann-Whitney rank sum tests. All other variables were categorical. Multiple linear regression models were used to seek correlations between continuous and categorical variables.

## Results

### Undifferentiated Thp-1 cells present several advantages to the commonly used differentiated Thp-1 cells assay

Several advantages were found associated with the uThp-1 assay compared to the commonly used dThp-1 assay, as summarized in table 1. This assay was faster, and specifically measured Fc-receptor mediated phagocytosis. Phagocytosis performed by uThp-1 varied in proportion to the amount of antibody bound to the target (figures 1A and 1B), and increased exponentially, following a sigmoidal curve, once the data were transformed. Consistency of results between different assays, on different days was high (figure 1C): intra-assay variability was minimal, with linear correlation between triplicates of serum samples run in the same day always high (r^2^>0.98).

**Table 1:** Main differences between new phagocytosis method and the common method used.

New assay	Commonly used assay
Undifferentiated Thp-1 assay	Differentiated Thp-1
Cells plated and used in the same day	Cells plated, stimulated and used 3 days later
Cells in suspension	Cells are adherent
56 Samples per 1 day/Assay (triplicate)	36 Samples per 3 day/Assay (triplicate)
Fluorescence based Assay – FACS	Colorimetric based assay- Elisa reader
Read-out: Percentage of phagocytic cells that have ingested labelled pRBC	Read-out: Amount of haemoglobin released by lysis of phagocytic cells
Possibility of including other parameters (e.g. cell surface markers)	Only one parameter measured
Phagocytic cells readily accessible for further studies	Phagocytic cells destroyed
Phagocytosis almost exclusively via Fc-receptors	Phagocytosis through various receptors/mechanisms

Comparison between phagocytosis assay done using undifferentiated Thp-1 cells (used in this study) and chemically- differentiated Thp-1 cells.

### Phagocytic antibodies' function in this cohort revealed the largest value spread and correlates with total amount of IgG to CS2 VSA

**Figure 2:**
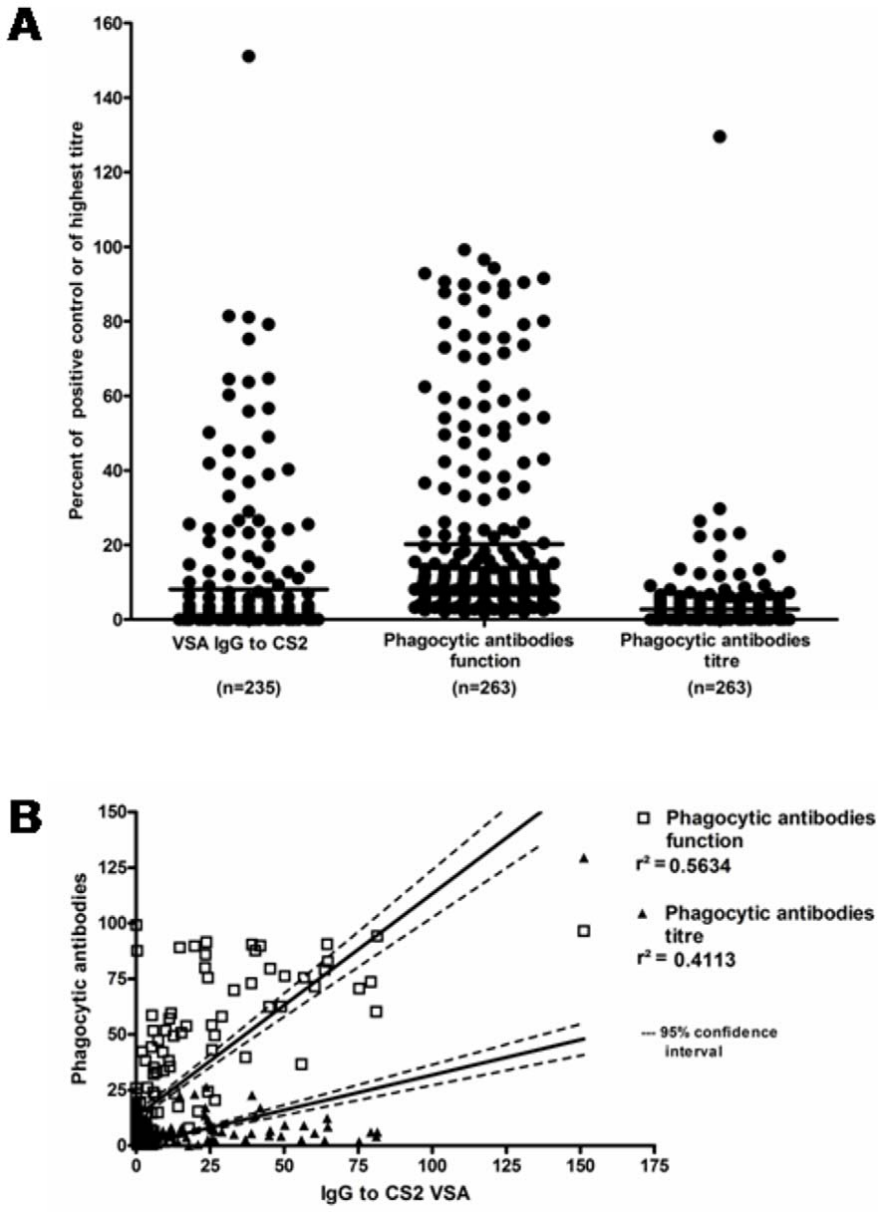
Distribution of, and correlation between, antibody parameters measured. A) Dot plot representation of the distribution of values obtained for VSA IgC to CS2, and both phagocytic antibodies' parameters. n =  number of samples analysed in each group. B) linear regression analysis of VSA IgG to CS2 and both function (open squares) and titre (full triangles) of phagocytic antibodies. Analysis performed on GraphPad Prism v4.0

Both the titre and the function of phagocytic antibodies correlated with levels of IgG to CS2 VSA (Spearman's rank correlation rho = 0.48 and 0.50 respectively, *p*<0.0001) but, when a linear regression was performed, correlation between function of phagocytic antibodies and IgG to CS2 VSA (r^2^ = 0.56) was stronger than the one found between titres and IgG to CS2 VSA (r^2^ = 0.41) owing to their spread over a larger range of values (figure 2A and 2B). For further analysis, we used the function of the phagocytic antibodies and referred to the titre only where significant differences between the two parameters occurred.

### HIV and *P. falciparum* infections are independently associated with maternal haemoglobin levels, maternal anaemia, infant birth weight and low birth weight

In a univariate analysis (table 2), both HIV and *P. falciparum* infection significantly decreased maternal haemoglobin levels and infant birth weight. The age of the women was significantly associated with both *P. falciparum* infection (being higher in uninfected women) and HIV (being higher in infected women). Women that were co-infected with HIV and *P. falciparum* had lower birth weight babies and presented with lower levels of haemoglobin than other groups (table 2).

**Table 2:** Associations of HIV and malaria infections in the cohort.

		Age			Maternal Hb level (g/dL)^1^			Infant Bw (g)^2^		
		n [%]	Mean (SD)	p-value	n [%]	Mean (SD)	p-value	n [%]	Mean (SD)	p-value
Entire Cohort		263 [100]	20.0 (3.3)		258 [100]	11.3 (1.98)		224 [100]	2904 (444)	
HIV	negative	156 [60]	19.5 (2.7)		155 [60]	11.6 (1.96)		123 [55]	2982 (413)	
	positive	106 [40]	20.7 (3.9)	*0.007*	102[40]	10.8 (1.93)	*0.002*	100 [45]	2808 (466)	*0.003*
Malaria infection	negative	104 [41]	20.7 (3.6)		152 [60]	11.5 (1.91)		101 [45]	2978(416)	
	positive	151 [59]	19.6 (2.7)	*0.0075*	98 [40]	10.9 (2.00)	*0.013*	121[54]	2845(464)	*0.016*
	Histology not present	8 [3]								
HIV and Malaria:										
	DOUBLE NEGATIVES^3^	43 [16]	19.6 (2.8)		43 [16]	12.1 (1.66)		40 [18]	3158 (402)	
	DOUBLE POSITIVES^4^	42 [16]	19.8 (3.0)	0.77	42 [16]	10.3(1.96)	*<0.0001*	41 [18]	2726 (559)	*0.0001*

Data represents means (standard deviation from means), and numbers of subjects [percent of total for each parameter].

1 Maternal haemoglobin levels.

2 Birth weight of babies at delivery.

3 DOUBLE NEGATIVES are women with neither HIV nor malaria.

4 DOUBLE POSITIVES are women with both HIV and malaria infection. Variables are normally distributed, so Student's *t-tests* were applied. Variables were analysed for all subjects for whom the relevant data were recorded.

When age, HIV status and *P. falciparum* malaria status were included in a multivariate analysis, both haemoglobin levels (HIV: coef = −1.1, *P* = <0.0001; malaria: coef = −0.8, *P* = 0.002) and birth weight (HIV: coef = −238.7, *P* = <0.0001; malaria: coef = −193.7, *P* = 0.002) were decreased by both infections.

With each of the infections, there was an increase in the prevalence of anaemia (HIV: coef = 0.19, *P* = <0.003; malaria: coef = 0.21, *P* = 0.001) and LBW (HIV: coef = 0.10, *P* = <0.06; malaria: coef = 0.10, *P* = 0.028), although the significance of the LBW prevalence increase with HIV was marginal.

### IgG to CS2 VSA and phagocytic antibodies are increased in *P. falciparum* -infected women and decreased in HIV-infected women

In a univariate analysis (Mann-Whitney ranksum), both phagocytic antibodies (Phago atb) and IgG to CS2 VSA (Total IgG) were higher in women with *P. falciparum* infection (Phago atb: z = 6.08, *P*<0.0001; Total IgG: z = 4.83, *P*<0.0001) and lower in HIV infected women (Phago atb: z = −3.75, *P* = 0.0002; Total IgG: z = −2.70, *P* = 0.007). Maternal haemoglobin levels and infant birth weights were not found to be associated with changes of IgG to CS2 VSA or phagocytic antibodies (table 3), and neither were the outcomes anaemia and LBW (data not shown).

**Table 3:** Association between indicated characteristics and PiAs function or IgG to CS2 VSA.

	Phagocytic function			Univariate analysis 1		Multivariate analysis^2^	
Variables		n	%	Z or Coeff (95% CI)	*P*	Coeff (95% CI)	*P*
	TOTAL	263	100				
Plac malaria							
	Negative	104	39.54				
	Positive	151	57.41	6.08	*<0.0001*		NA
	Histology not present	8	3.04				
HIV							
	Negative	157	59.70				
	Positive	106	40.30	−3.75	*0.0002*	−10.97 (−21.33, −0.61)	*0.038*
Hemoglobin levels^3^	Present	261	99.24	−7.31 (−2.25, 0.781)	0.342	−0.99 (−3.36, 1.39)	0.412
	Unknown	2	0.76				
Infant Birthweight^3^	Present	227	86.31	−0.004 (−0.011, 0.03)	0.227	0.001 (−0.009, 0.012)	0.834
	Unknown	36	13.69				

	IgG to CS2 VSA			Univariate analysis		Multivariate analysis	
Variables		n	%	Z or Coef (95% CI)	*P*	Coefficient (95% CI)	*P*
	TOTAL	234	100				
Plac malaria							
	Negative	91	38.89				
	Positive	137	58.55	4.83	*<0.0001*		NA
	Histology not present	6	2.56				
HIV							
	Negative	141	60.26				
	Positive	93	39.74	−2.74	*0.0007*	−7.60 (−16.9, 1.68)	0.107
Hemoglobin levels	Present	233	41.45	−0.455 (−1.72, 0.80)	0.477	−0.56 (−2.90, 1.78)	0.637
	Unknown	1	0.43				
Infant Birthweight	Present	202	10.68	−0.003 (−0.009,0.004)	0.400	0.002 (−0.008, 0.012)	0.708
	Unknown	32	13.68				

1 Function of phagocytic antibodies (top) and IgG to CS2 VSA (bottom) unadjusted analysis (Mann-Whitney Ranksum for HIV and Malaria infections with Z-statistic score and Linear Regression analysis for Maternal haemoglobin levels and Infant birth weight with coefficients and 95% confidence intervals. P-values are also shown).

2 Adjusted estimate for malaria positive women only (Multi linear model correlation coefficients and respective 95% confidence intervals and *P*-values are shown). (n) number of women (NA) non-applicable (Z) Z-statistic (coeff (95% CI)) correlation coefficient and 95% confidence intervals. A negative Z-statistic or coefficient implies a decrease of antibody levels. A positive Z-statistic or coefficient implies an increase in antibody levels.

In a multivariate analysis, using multiple linear regression methods, on *P. falciparum* positive women, HIV infection was associated with decreased phagocytic antibodies, but neither maternal haemoglobin levels, infant birth weight nor HIV impacted on IgG to CS2 VSA (table 3).

When the multivariate analysis included both *P. falciparum*-positive and *P. falciparum* -negative women both IgG to CS2 VSA and phagocytic antibodies were found to be associated with HIV (Table S1). No associations were found between HIV positivity and antibody levels in *P. falciparum*-negative women only (data not shown).

### IgG to CS2 VSA and function of phagocytic antibodies across the different histopathology groups are differently modulated by HIV infection

**Figure 3:**
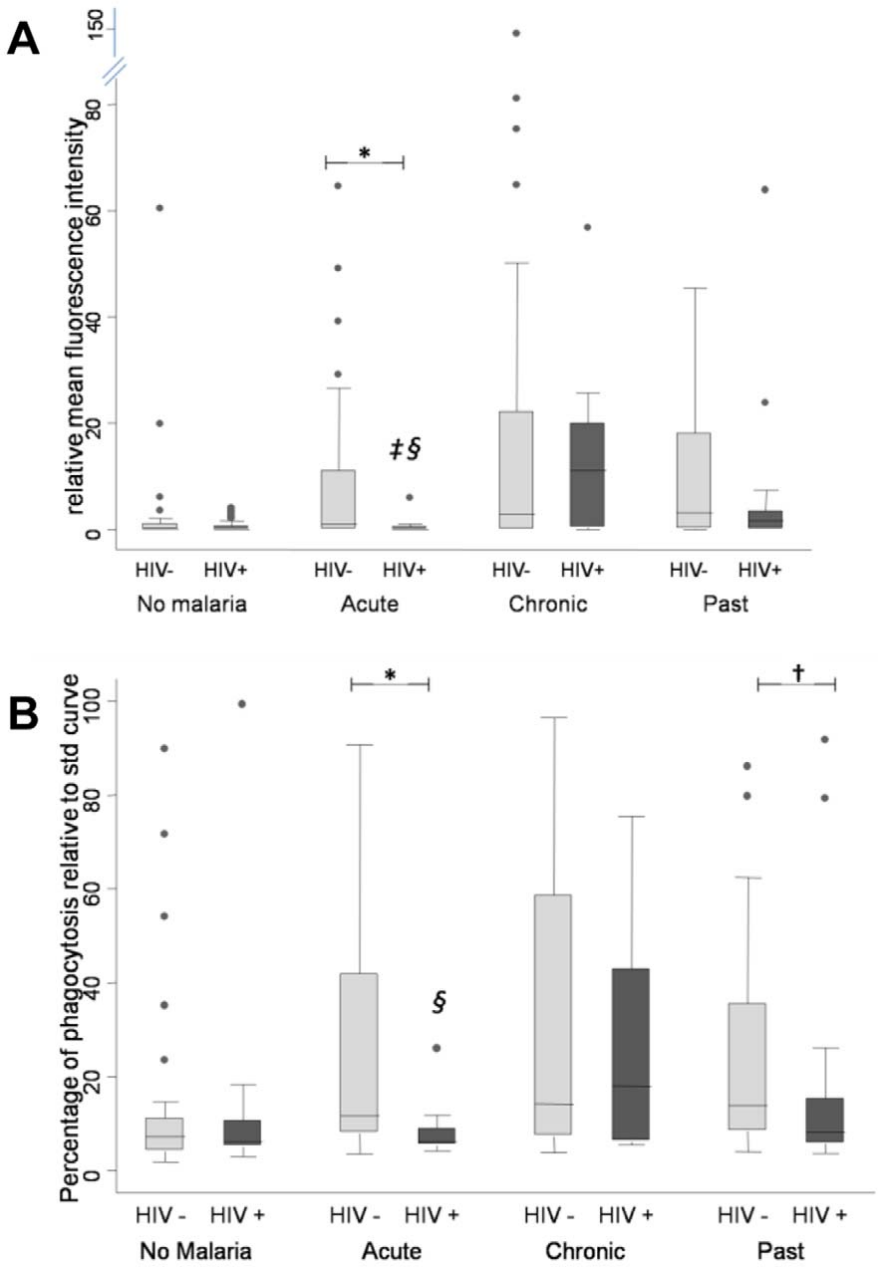
Antibodies across histopathology groups of placental malaria. Antibody measurements across histopathology of placental malaria groups. Light grey bars – HIV negative (HIV−), dark grey bars – HIV positive (HIV = ). A) Total VSA IgG to CS2. Mann-Whitney ranksum tests performed on HIV− women: §acute vs chronic *p* = 0.018, ‡ acute vs past: *p* = 0.038. Mann-Whitney ranksum tests performed between HIV− and HIV+ women: * acute group: *p* = 0.028. B) phagocytic antibodies' function. Mann-Whitney ranksum tests performed on HIV- women: § acute vs chronic *p* = 0.034. Mann-Whitney ranksum tests performed between HIV− and HIV+ women: * acute group: *p* = 0.013, † past group: *p* = 0.07. Bars represent median and interquartile ranges. Whiskers represent 95% CI. Dots represent outliers.

When analysis of antibody measurements was made according to the placental malaria status of the women, similar levels of both VSA IgG and phagocytic antibodies were observed in the groups with current or previous *P. falciparum* infection and no HIV (figure 3, light grey bars).

When HIV positive women were analysed with regard to their antibody measurements (figure 3, dark grey bars), women in the acute infection group had significantly lower IgG to CS2 VSA levels than women in the combined chronic and past groups (Mann-Whitney ranksum: *p = *0.018 and *p = *0.038, respectively, figure 3A) and significantly lower phagocytic antibodies function than women in the chronic group (Mann-Whitney ranksum: *p = *0.034, figure 3B).

The comparison between antibody measurements in HIV positive and HIV negative women in the different histopathology groups revealed that HIV positive women in the acute group failed to generate antibodies to pRBC, both total (Mann-Whitney ranksum: *p = *0.028, figure 3A) and phagocytic (Mann-Whitney ranksum: *p = *0.013, figure 3B), when compared to the HIV negative women. HIV positive and negative women in the chronic infection group had similar levels of antibodies. HIV infected women in the past infection group had lower levels of phagocytic antibodies than did HIV negative women in this group (Mann-Whitney ranksum: *p = *0.07).

Peripheral blood CD4 T-cell counts appeared to be correlated with levels of antibody to pRBC. HIV positive women who presented with a CD4-T cell count less than 350×10^6^ cells/L had lower levels of IgG to CS2 VSA (coef: −6.15, P = 0.007) and less phagocytic antibodies (coef: −11.95,P = 0.002) than did HIV infected women with CD4 T-cell counts above 350×10^6^ cells/L.

## Discussion

Undifferentiated Thp-1 cells had previously been shown to be useful when measuring erythrophagocytosis [27], but no high-throughput assay concerning Fc-mediated phagocytosis that could actually be used consistently on large sample sets existed. In this study an improved, high-throughput method has been developed in order to more accurately and rapidly estimate Fc-receptor mediated phagocytosis induced by *P. falciparum* malaria-specific antibodies. Such a method facilitates the measurement of functional antibodies to malaria VSA in large sample sets. Based on the use of undifferentiated Thp-1 cells that grow in suspension, this assay lends itself to various small modifications that might be used to study both the effector cell response to pRBC expressing specific ligands, and the antibody responses to these pRBC. In comparison with the dThp-1 cells assay that has been more widely used [11], [14], [21], there are various advantages (table 1). The use of standard curves allows the immediate comparison of the behaviour of the uThp-1 across different experimental days and also is an assurance of inter-assay consistency of results. Undifferentiated Thp-1 cells are also of particular interest in the study of phagocytic antibodies since it is clear that the IgG-mediated phagocytosis performed by these cells is through Fcγ receptors I and II, the only Fcγ receptors present in these cells [17]. Of note, uThp-1 cells do not express CD36 [19] a known cell-membrane receptor that can promote phagocytosis of unopsonised pRBC [20]. uThp-1 cells were unable to phagocytose unopsonised pRBC of the CD36-binding parasite line 3D7, and also, although uThp-1 express CD54 (ICAM-1), the CD54-binding parasite line E8B-ICAM did not trigger a phagocytic response in the absence of immune serum (authors' unpublished data), demonstrating that this cytoadherence ligand is not involved in uThp-1-mediated phagocytosis. The assays reported here were performed using GFP expressing parasites and uThp-1 cells, but the assay can be adapted to use EtBr stained parasites and positively selected donor monocytic cells (authors' unpublished data), which permit the measurement of responses against multiple parasite lines, and to study variation in host phagocytic cell responses.

Levels of IgG to CS2 VSA as well as function elicited by phagocytic antibodies had a larger spread than the actual titre of the phagocytic antibodies observed, and we postulate that this is due to the limited exposure of primigravidae in this study to pregnancy specific parasites. In cohorts of multigravid women, with greater histories of exposure and more developed pregnancy-specific immunity, titres of phagocytic antibodies may have a larger spread, and may thus be more informative. The choice of function or titre for analysis is dependent on the research question, though both parameters can provide valuable information.

Both HIV and malaria were strongly and independently associated with the occurrence of low maternal haemoglobin and lower birth weight babies, and as expected, co-infected women and their babies had the worst outcomes [9], [28]. Levels of IgG to CS2 VSA and phagocytic antibodies were lower in HIV positive women when compared to HIV negative women and higher in women with malaria infection when compared to women without malaria infection. In our cohort of primigravidae, antibodies were not found to be associated with protection from anaemia or LBW (known to be important outcomes associated with placental malaria (reviewed in [3], [29]), instead antibodies reflect mainly exposure to antigen. Similar results, with a lack of association between adverse outcomes and presence of antibodies to malaria in pregnancy related antigens, have recently been published [30]. It may be possible that a certain titre and “quality” of such antibodies has to be present in circulation at very early stages during the pregnancy for a protective effect (or an association with one) to be observed, something already suggested by other studies [7], [30], [31]. In a multivariate analysis including HIV, maternal haemoglobin level and infant birth weight, using the malaria infected women, HIV was found to be associated with a decrease of phagocytic antibodies function but not of levels of IgG to CS2 VSA. A similar result with HIV affecting phagocytic antibodies function (opsonic-activity) but not other types of antibody was obtained by Jaworowski et al, using multigravid women from this same study cohort [11]. HIV may therefore impact differently on the production of functionally distinct antibodies (as suggested also by [15]).

When the women were grouped according to their placental malaria status, measures of the function of phagocytic antibodies and the total IgG to CS2 VSA tended to vary between current, chronic and past placental infection, although differences only reached statistical significance in HIV positive women. Antibody levels were highest in the women with chronic infection (as observed by others [6]), supporting the view that constant exposure to the parasite increases production of antibody. Unlike the previous study, which included women of all gravidities, we focussed solely on primigravidae, indicating that in a single pregnancy, chronic infection leads to strong anti-VSA antibody responses. In the absence of HIV infection, the levels of antibody in acute infection were very similar to those in chronic infection, suggesting that robust antibody responses to these antigens can develop rapidly, as observed also in other studies [31]. Further investigation of the relationship between placental histopathology, duration of infection and levels of antibodies in HIV positive versus HIV negative women would be of significant interest in confirming the differences we have observed.

Grouping according to placental malaria status provides also a putative timeline of exposure to pregnancy specific antigens and of antibody acquisition: Women with No Malaria may have had little or no exposure to antigen. In Acute Infection, antibody responses are developing, following recent exposure to antigen. Chronic Infection represents repeated exposure to antigen, and antibody maturation and/or recall, and Past Infection is associated with antibody maintenance, following past exposure to antigen. This is in keeping with established paradigms of the time course of placental infection [32].

This classification seems to suggest that HIV infection reduced the generation of antibodies and possibly affected the maintenance of antibodies after clearance of malaria infection, when comparing HIV-positive with HIV-negative women. The time-frame of one pregnancy is probably not ample enough to fully assess the impact that HIV can have on the maintenance of antibody repertoires, but it is well established that HIV negatively influences memory B cells, impairing the long-term serological memory of the infected individuals [33], [34]. Future studies looking at the levels of these same antibodies 6 months after delivery or in subsequent pregnancies in HIV-negative and HIV- positive women and comparing numbers and activity of memory B-cells may prove to be more informative.

In the chronic infection group, HIV did not seem to impact on the level of IgG to CS2 VSA or function of these antibodies, suggesting that greater exposure may overcome the impairment in antibody production elicited by HIV infection. Despite levels of antibody that may seem sufficient, HIV infected women may lack, or have impaired, phagocytic cells that would respond to these antibodies, making the evaluation of the capacity of these women's effector cells in responding to opsonised targets a major area of interest.

In summary, a new assay to measure the activity of phagocytic antibodies was developed and revealed to be high throughput and reproducible, making it ideal to investigate these antibodies in large sample sets. By applying the assay to a cohort of primigravid women, which are the most susceptible group to suffer from the deleterious effects of placental malaria, we have shown that primigravidae can exhibit a strong antibody response to pregnancy-specific parasites which can be measured through the use of their serum to opsonise and subsequently promote the uptake of CS2 pRBC. These antibodies correlate with exposure to pregnancy-specific parasites and with levels of IgG to VSA of the placenta-like parasite strain CS2, although these responses do not appear to protect against complications of pregnancy malaria in first pregnancy. By grouping the women according to a putative time-line of exposure to the parasite we were able to see the effect that HIV has on the acquisition of antibodies and, possibly, on their maintenance. Interestingly, in a multivariate analysis of malaria positive women, HIV infection significantly impaired phagocytic antibodies but not levels of IgG to CS2 VSA. It is unusual for studies to focus only on primigravid women due to their limited range of antibodies. Despite that fact, in this study our protocol allowed us not only to measure biologically functional antibodies, but also relate them to HIV status and even placental malaria status. From this, we conclude that women in their first pregnancy are able to mount a phagocytic antibody response to pregnancy specific malaria parasites that may be important for subsequent pregnancies, and that this response can be severely modulated in the presence of HIV. These findings may prove to be important in the context of developing vaccines towards malarial antigens in areas where HIV has a considerable impact.

## Supporting Information

Table S1 Multivariate analysis on all women of the association between indicated characteristics and PiAs function or IgG to CS2 VSA. Function of phagocytic antibodies (top) and IgG to CS2 VSA (bottom) adjusted estimates for malaria positive and negative women (Multi linear model correlation coefficients and respective 95% confidence intervals and P-values are shown). (n) number of women (NA) non-applicable (coeff (95% CI)) correlation coefficient and 95% confidence intervals. A negative coefficient implies a decrease of antibody levels. A positive coefficient implies an increase in antibody levels.
(0.06 MB DOC)
