# Supplementary material for: Using an Improved Phagocytosis Assay to Evaluate the Effect of HIV on Specific Antibodies to Pregnancy-Associated Malaria
Source: PLoS One. 2010 May 25;5(5):e10807. doi: 10.1371/journal.pone.0010807 (PMC2876038; doi:10.1371/journal.pone.0010807)
Supplement: Table S1 — Multivariate analysis on all women of the association between indicated characteristics and PiAs function or IgG to CS2 VSA. Function of phagocytic antibodies (top) and IgG to CS2 VSA (bottom) adjusted estimates for malaria positive and negative women (Multi linear model correlation coefficients and respective 95% confidence intervals and P-values are shown). (n) number of women (NA) non-applicable (coeff (95% CI)) correlation coefficient and 95% confidence intervals. A negative coefficient implies a decrease of antibody levels. A positive coefficient implies an increase in antibody levels. (0.06 MB DOC) [file pone.0010807.s001.doc]

**Table S1 – Multivariate analysis on all women of the association between indicated characteristics and PiAs function or IgG to CS2 VSA**.

|  |  |  |  | Phagocytic function |  |
| --- | --- | --- | --- | --- | --- |
| Variables |  | n | % | Coeff (95% CI) | *P* |
|  |  |  |  |  |  |
|  | TOTAL | 263 | 100 |  |  |
|  |  |  |  |  |  |
| Placental malaria |  |  |  |  |  |
|  | Negative | 104 | 39.54 |  |  |
|  | Positive | 151 | 57.41 |  | NA |
|  | Histology not present | 8 | 3.04 |  |  |
| HIV infection |  |  |  |  |  |
|  | Negative | 157 | 59.70 |  |  |
|  | Positive | 106 | 40.30 | -11.34 (-17.60, -5.08) | <0.0001 |
|  |  |  |  |  |  |
| Haemoglobin levels^3^ | Present | 261 | 99.24 | -1.38 (-2.96, 0.19) | 0.084 |
|  | Unknown | 2 | 0.76 |  |  |
|  |  |  |  |  |  |
| Infant Birthweight^3^ | Present | 227 | 86.31 | -0.004 (-0.01, -0.002) | 0.194 |
|  | Unknown | 36 | 13.69 |  |  |
|  |  |  |  |  |  |
|  |  |  |  | IgG to CS2 VSA |  |
| Variables |  | n | % | Coeff (95% CI) | *P* |
|  |  |  |  |  |  |
|  | TOTAL | 234 | 100 |  |  |
|  |  |  |  |  |  |
| Placental malaria |  |  |  |  |  |
|  | Negative | 91 | 38.89 |  |  |
|  | Positive | 137 | 58.55 |  | NA |
|  | Histology not present | 6 | 2.56 |  |  |
| HIV |  |  |  |  |  |
|  | Negative | 141 | 60.26 |  |  |
|  | Positive | 93 | 39.74 | -8.20 (-13.40, -3.01) | *0.002* |
|  |  |  |  |  |  |
| Haemoglobin levels^3^ | Present | 233 | 41.45 | - 0.74 (-2.10, 0.62) | 0.282 |
|  | Unknown | 1 | 0.43 |  |  |
|  |  |  |  |  |  |
| Infant Birthweight^3^ | Present | 202 | 10.68 | -0.002 (-0.008, 0.004) | 0.453 |
|  | Unknown | 32 | 13.68 |  |  |
|  |  |  |  |  |  |

Function of phagocytic antibodies (top) and IgG to CS2 VSA (bottom) adjusted estimates for malaria positive and negative women (Multi linear model correlation coefficients and respective 95% confidence intervals and *P*-values are shown). (n) number of women (NA) non-applicable (coeff (95% CI)) correlation coefficient and 95% confidence intervals. A negative coefficient implies a decrease of antibody levels. A positive coefficient implies an increase in antibody levels.
